# Integrative GC-MS, network pharmacology, and molecular dynamics elucidate synergistic anti-diabetic mechanisms of Chongqing *Citrus reticulata* ‘Dahongpao’ volatile oil via multi-target stabilization

**DOI:** 10.1371/journal.pone.0338723

**Published:** 2026-01-16

**Authors:** Wanting Zhong, YaYi Xiong, Jie Luo, Shujaat Ahmad, Jian Wang

**Affiliations:** 1 College of Traditional Chinese Medicine, Chongqing Medical University, Chongqing, China; 2 Department of Pharmacy, Shaheed Benazir Bhutto University, Sheringal, Dir Upper, Pakistan; 3 School of Chinese Materia Medica, Chongqing University of Chinese Medicine, Chongqing, China; Government College University Faisalabad, PAKISTAN

## Abstract

**Background:**

Diabetes mellitus involves complex pathogenesis requiring multi-target interventions. *Citrus reticulata* ‘Dahongpao’ from Chongqing exhibits anti-diabetic potential, but its mechanisms remain elusive.

**Methods:**

We employed an integrative strategy: GC-MS identified 82 compounds (96.61% coverage), dominated by *D*-limonene (62.48%). Network pharmacology revealed 36 diabetes-related targets. Molecular docking prioritized ligands (thymol: −6.8 kcal/mol with FABP1; n-hexadecanoic acid: −6.7 kcal/mol with PTGS2). Critical validation was achieved via 100-ns molecular dynamics (MD) simulations and MM-GBSA binding free energy calculations.

**Results:**

MD simulations demonstrated structural stability (RMSD < 2.5 Å) for core complexes (e.g., CYP19A1/thymol). MM-GBSA quantified robust binding for FABP1/dodecanoic acid (−43.26 kcal/mol) and PTGS2/n-hexadecanoic acid (−43.93 kcal/mol), driven by van der Waals forces. Hydrogen bond dynamics revealed persistent interactions (e.g., thymol–THR102 in FABP1), while RMSF highlighted ligand-induced flexibility in fatty acids. Pathway analysis implicated PPAR signaling and insulin resistance.

**Conclusion:**

*Citrus reticulata* ‘Dahongpao’ essential oil combats diabetes through synergistic multi-target modulation, validated by dynamic ligand–protein stability and energetics. This study presents an in silico framework that integrates phytochemical profiling and computational analyses to facilitate natural product drug discovery.

## Introduction

Diabetes mellitus is a globally prevalent chronic metabolic disease characterized by a complex pathogenesis that primarily includes insulin resistance, inflammatory responses, and oxidative stress [1]. According to the Flora of China, ‘Dahongpao’ is a variety of red tangerine classified under the phylum Angiospermae, family Rutaceae, and genus *Citrus* [2]. The peel of Hongju, which is the pericarp of the fruit, is a traditional Chinese medicinal material. Its essential oil components are believed to have potential applications in the treatment of diabetes. The purpose of this study is to explore and elucidate the antidiabetic mechanisms of *Citrus reticulata* ‘Dahongpao’ peel essential oil through multiple technical approaches.

According to recent research, the essential oil of *Citrus reticulata* ‘Dahongpao’ produced in Bishan, Chongqing mainly consists of monoterpenes, sesquiterpenes, and their oxygenated derivatives. These components exhibit significant biological activities, including antioxidant, anti-inflammatory, blood glucose–regulating, and lipid metabolism–modulating effects. Specifically, a study by Wang Jian and Wang Gang indicated that the essential oil of *Citrus reticulata* ‘Dahongpao’ peel from Bishan, Chongqing, mainly comprises monoterpenes, sesquiterpenes, and their oxygenated derivatives, which show notable bioactivities in antioxidation, anti-inflammation, glycemic regulation, and lipid modulation [3]. Among them, limonene is the most abundant constituent, accounting for as much as 48.089% of the oil. Other key components include linalool, thymol, γ-terpinene, and α-sinensal. These compounds demonstrate remarkable biological effects. For example, linalool, a major alcohol in the essential oil of Hongju peel, has shown significant antidiabetic activity. It enhances cellular glucose uptake and utilization by activating the AMPK signaling pathway and increasing the expression of glucose transporter GLUT4 [4], thereby directly helping to alleviate hyperglycemia in diabetic patients. Current studies at home and abroad have found that thymol possesses antioxidant, free radical–scavenging, anti-inflammatory, analgesic, antispasmodic, antibacterial, antifungal, preservative, and antitumor activities [5,6]. Additionally, γ-terpinene, one of the primary active components in cumin (Cuminum cyminum) essential oil, has therapeutic effects on diabetes, including reducing serum levels of glycated hemoglobin, triglycerides, and cholesterol, while increasing insulin levels [7].

Network pharmacology is an emerging drug development strategy that combines systems biology and computational biology methods to explore the interactions between complex diseases and drugs. This approach has attracted considerable attention in the field of traditional medicine, as it reveals how drug molecules act on multiple targets and pathways involved in complex disease processes. In recent years, network pharmacology has been applied to analyze the potential mechanisms of action of traditional herbal medicines and extracts, including essential oils. For complex mixtures like *Citrus reticulata* ‘Dahongpao’ essential oil, network pharmacology offers a comprehensive perspective to identify its key antidiabetic constituents and mechanisms of action.

Although specific research on *Citrus reticulata* ‘Dahongpao’ peel essential oil remains limited, this study integrates chemical analysis, network pharmacology, molecular docking, and molecular dynamics to conduct an in-depth investigation of its components and predict its potential therapeutic effects in diabetes treatment.

## Materials and methods

### Materials and instruments

Materials: Experimental material: Dried mature peel of *Citrus reticulata* ‘Dahongpao’ collected from Bishan, Chongqing, China. Identified by Professor Wang Jian (School of Traditional Chinese Medicine, Chongqing Medical University). Anhydrous sodium sulfate (analytical grade, CAS No. 7757-82-6, Greagent, 500 g) was used for dehydration. Nonane (purity 98%, CAS No. 111-84-2, Adamas Life, 100 mL) was used as an internal standard compound.

Instruments: Gas chromatography-mass spectrometry (GC-MS) system GC 7890B/5977B (Agilent Technologies, USA) equipped with a DB-5 capillary column (30.0 m × 0.32 mm × 0.5 μm). Bond A3 Pipette manual single-channel adjustable pipette (range: 10–100 µL, Titan). Finnpipette F3 2–20 µL Micro single-channel adjustable pipette (range: 2–20 µL, Thermo Scientific).

### Preparation and identification of essential oil

#### Preparation of essential oil.

According to the essential oil extraction method described in the Chinese Pharmacopoeia (2020 edition), and considering literature indicating that the relative density of *Citrus reticulata* ‘Dahongpao’ peel essential oil is less than 1, Method A for essential oil determination in the pharmacopoeia was adopted [8].

Dried *Citrus reticulata* ‘Dahongpao’ peels were stored in a cool, dry place. A total of 200 g of peel was weighed, cut into small pieces, and placed into a 2000 mL round-bottom flask. Then, 1200 mL of purified water was added. The flask was connected to an essential oil extractor and heated with a heating mantle until a gentle boil was reached. The initial extraction lasted for 4 hours, after which the mixture was cooled and the essential oil was collected in a dry, sealed container. This process was repeated twice more (each time 4 hours), and the collected essential oils were combined. To remove any remaining moisture, a small amount of anhydrous sodium sulfate was added for dehydration over 24 hours. The solution was then filtered and stored in screw-cap vials in a refrigerator at 4°C.

#### Identification of essential oil.

GC-MS analysis was performed using an Agilent GC 7890B/5977B system equipped with a DB-5 capillary column (30.0 m × 0.32 mm × 0.5 μm). Analytical conditions for GC-MS were as follows: carrier gas: high-purity helium (99.999%) at a flow rate of 1.0 mL·min ⁻ ¹; injection volume: 1.0 μL; injector temperature: 260°C; transfer line temperature: 260°C; split ratio: 20:1; ionization mode: electron impact (EI); electron energy: 70 eV; ion source temperature: 200°C; mass scan range (m/z): 10–2000 amu; scan rate: 3.9 scans·s ⁻ ¹.

The oven temperature program was: initial temperature at 60°C (held for 3 min), ramped at 5°C/min to 240°C, and held for 6 min.

Compound identification was based on total ion chromatograms (TIC), with background subtraction. The mass spectra were compared with entries in the NIST database or commercially available standards (e.g., C7–C40 n-alkane standard solution). For overlapping peaks, the Automated Mass Spectral Deconvolution and Identification System (AMDIS) was used for separation and purification, followed by identification based on retention indices. All samples were standardized by adding a fixed amount of internal standard compound (e.g., nonane).

For quantification, peak area normalization was first used to calculate the relative content of each compound. Then, by measuring the response factor of each characteristic component relative to the internal standard (nonane), the concentration of each identified peak in the essential oil was quantified.

The raw GC-MS data were processed using Agilent MassHunter Workstation software (Qualitative Analysis B.08.00). Metabolites were characterized using the NIST.14 database. Data were then normalized and processed using Excel to compile compound names, retention times, peak intensities, and mass values for all samples.

### Network pharmacology

As the medicinal material used in this study is a local specialty from Chongqing and not listed in standard references such as the Pharmacopoeia, traditional databases like TCMSP could not be used for screening the constituents of “*Citrus reticulata* ‘Dahongpao’ peel.” Therefore, based on previous studies, the chemical constituents of *Citrus reticulata* ‘Dahongpao’ peel were first identified via GC-MS and verified with the NIST database and Professor Wang Jian. These constituents were then individually searched on PubChem [9], and their chemical structures were drawn using PubChem and ChemDraw.

Next, SwissADME was used to screen for bioactive compounds [10]. The screening criteria were: gastrointestinal index (GI) absorption = “High,” blood–brain barrier (BBB) permeant = “Yes,” and at least two “Yes” entries under drug-likeness filters. Compounds meeting these criteria were considered potential bioactive ingredients. The structures of these candidate compounds were then input into SwissTargetPrediction [11] to obtain their potential targets. After compiling all potential active compounds and their predicted targets, targets with a probability score < 0.1 were excluded. The remaining targets were validated using UniProt to ensure they were drug-related.

Cytoscape software was used to construct the compound–target network diagram [12]. Disease targets were retrieved from GeneCards [13] using the keyword “Diabetes.” The median value from three separate searches was used to screen relevant disease targets. The intersection of compound and disease targets was determined using the Bioinfogp website [14] and visualized as a Venn diagram.

Protein–protein interaction (PPI) analysis of the overlapping targets was performed using the STRING database [15]. Core targets were screened based on topological parameters: degree, betweenness centrality, and closeness centrality. These intersecting targets were then uploaded to the DAVID platform for functional enrichment analysis [16], using “Homo sapiens” as the selected species to obtain Gene Ontology (GO) terms and Kyoto Encyclopedia of Genes and Genomes (KEGG) pathways. The results were visualized using the “Microbiology Letter” (WeiShengXin) platform [17].

### Molecular docking

Molecular docking was performed between the core targets obtained from network pharmacology and the selected small-molecule compounds. Protein structures of the core targets were downloaded from the Protein Data Bank (PDB), while the small-molecule structures were obtained from PubChem in SDF format. Docking was carried out using AutoDock Vina 1.1.2. The receptor and ligand molecules were pre-processed, optimized, and minimized prior to docking. The docking grid covered the entire protein using semi-flexible docking.

Visualization of selected docking results was performed using PyMOL 2.1 software to analyze the binding modes of compounds with their target proteins, including hydrogen bonding interactions. Based on binding energy values and molecular interactions, the binding affinities of the screened compounds were evaluated.

### Molecular dynamics

All-atom molecular dynamics (MD) simulations were performed using the AMBER 22 software package [18], with the initial structures derived from the previously obtained protein–ligand docking complexes. Prior to simulation, the atomic partial charges of the small molecules were calculated using the antechamber module and Hartree–Fock (HF) SCF/6-31G* level of theory via Gaussian 09 software [19,20]. The small molecules and proteins were parameterized using the GAFF2 and ff14SB force fields, respectively [21,22].

System preparation was carried out using the LEaP module, in which hydrogen atoms were added, and each complex was solvated in a truncated octahedral TIP3P water box extending 10 Å from the solute boundary [23]. Sodium and chloride ions (Na ⁺ /Cl⁻) were added to neutralize the system. The topology and coordinate files were then generated for the subsequent MD steps.

Energy minimization was performed in two stages: 2500 steps of steepest descent followed by 2500 steps of conjugate gradient minimization. The minimized system was gradually heated from 0 K to 298.15 K over 200 ps under constant volume conditions. This was followed by a 500-ps NVT (constant volume and temperature) simulation at 298.15 K to allow uniform solvent distribution, and an additional 500-ps NPT (constant pressure and temperature) equilibration.

The production MD was then run for 100 ns under periodic boundary conditions in the NPT ensemble. A non-bonded interaction cutoff of 10 Å was applied. Long-range electrostatic interactions were computed using the Particle Mesh Ewald (PME) method [24], while bond lengths involving hydrogen atoms were constrained using the SHAKE algorithm [25]. Temperature control was maintained using the Langevin thermostat with a collision frequency (γ) of 2 ps ⁻ ¹ [26]. The pressure was maintained at 1 atm, with an integration time step of 2 fs, and trajectory snapshots were saved every 10 ps for further analysis.

The binding free energies between proteins and ligands in all systems were estimated using the Molecular Mechanics/Generalized Born Surface Area (MM/GBSA) method [27–30]. As extended molecular dynamics trajectories may reduce the accuracy of MM/GBSA predictions [28], snapshots extracted from the last 10 ns (90–100 ns) of the simulations were used for energy calculations. The binding free energy was calculated according to the following equation:


ΔGbind=ΔGcomplex−(ΔGreceptor+ΔGligand)=ΔEinternal+ΔEVDW+ΔEelec+ΔGGB+ΔGGA

In this equation, ΔEinternal represents the internal energy contribution, including bond (Ebond), angle (Eangle), and torsional (Etorsion) energies. ΔEVDW and ΔEelec correspond to the van der Waals and electrostatic interactions, respectively. ΔGGB and ΔGSA are the polar and non-polar components of solvation free energy.

The polar solvation energy (ΔGGB) was computed using the Generalized Born (GB) model developed by Nguyen et al. (igb = 2) [31]. The non-polar solvation energy (ΔGSA) was estimated using the empirical relation ΔGSA = 0.0072 × SASA [32], where SASA is the solvent-accessible surface area and 0.0072 kcal/mol·Å² is the surface tension coefficient.

The entropy contribution (TΔS) was not included due to its high computational cost and relatively low accuracy in this context [27,28].

## Results and discussion

### Qualitative and quantitative analysis of volatile oil

The volatile oil from *Citrus reticulata* ‘Dahongpao’ was analyzed using GC-MS, with nonane as the internal standard for quantification. After a 66.66-minute detection period, the chromatogram was obtained as shown below.

**Fig 1:**
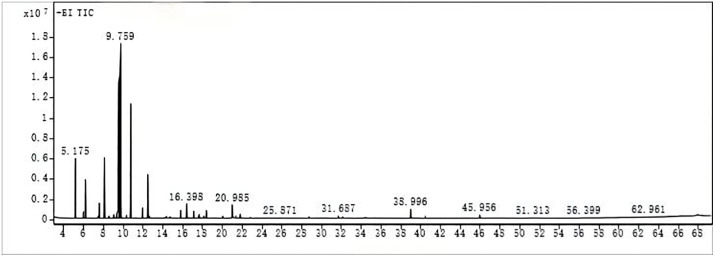
Chromatogram of the Volatile Oil.

From the Fig 1, the peak at 5.175 min corresponds to the internal standard (nonane). Excluding the internal standard, a total of 82 compounds were identified, accounting for 96.61% of the total peak area of the volatile oil. The major compound is *D*-limonene, a monoterpene with a lemon-like aroma commonly found in C*itrus* plants, which constitutes 62.48% of the total compound area. The second most abundant compound is γ-terpinene, also a monoterpene with strong volatility and fragrance, accounting for 9.82%. Linalool, a monoterpene alcohol with a floral scent found in plants like lavender and lemongrass, accounts for 3.37%. Additionally, β-myrcene, a monoterpene with an earthy aroma, accounts for 4.67%, and L-α-terpineol, a monoterpene alcohol with a pine-like scent, accounts for 1.19%. All these compounds belong to the monoterpene class, characterized by their high volatility and fragrance.

Further analysis revealed the presence of trace compounds such as α-pinene and camphene, both monoterpenes. Although present in small amounts, these compounds contribute significantly to the overall chemical composition and characteristics of the sample. Long-chain compounds such as n-hexadecanoic acid were also detected, indicating the presence of lipid components in the sample.

In terms of compound types, the sample is predominantly composed of monoterpenes, which account for the majority of the compound area and are the main components of *Citrus* volatile oils. Small amounts of monoterpene alcohols, such as linalool and L-α-terpineol, were also detected, which have strong aromatic and pharmacological properties.

*D*-limonene is one of the key characteristic compounds. β-myrcene is also present in significant amounts. Some of the detected components may be related to the extraction and processing methods, possibly containing impurities or intermediate products. Detailed chemical composition, retention times, retention indices, and related information are presented in the table below (Table 1).

**Table 1:** Qualitative and Quantitative Analysis of Volatile Oil Components.

NO.	Retention time (min)	Percentage of area (%)	Name of chemical composition	CAS	Retention index	Matching retention index
**1**	3.73	0.03	Furfural	98-01-1	835	833
**2**	5.98	0.39	Bicyclo [3.1.0]hex-2-ene, 2-methyl-5-(1-methylethyl	2867-05-2	930	929
**3**	6.19	2.54	α-Pinene	80-56-8	937	937
**4**	6.65	0.02	Camphene	79-92-5	951	951
**5**	7.47	0.17	Bicyclo [3.1.0]hexane, 4-methylene-1-(1-methylethyl)-	3387-41-5	976	974
**6**	7.58	1.12	β-Pinene	127-91-3	978	979
**7**	8.10	4.83	β-Myrcene	123-35-3	992	991
**8**	8.57	0.31	α-Phellandrene	99-83-2	1005	1005
**9**	9.05	0.43	1,3-Cyclohexadiene, 1-methyl-4-(1-methylethyl)-	99-86-5	1019	1017
**10**	9.55	64.67	*D*-limonene	5989-27-5	1032	1032
**11**	9.94	0.02	1,3,6-Octatriene, 3,7-dimethyl-, (Z)-	3338-55-4	1042	1038
**12**	10.33	0.22	trans-β-Ocimene	3779-61-1	1052	1049
**13**	10.76	10.16	γ-Terpinene	99-85-4	1062	1060
**14**	11.31	0.07	2-Furanmethanol, 5-ethenyltetrahydro-α,α,5-trimethyl-, cis-	5989-33-3	1075	1074
**15**	11.95	0.86	Cyclohexene, 1-methyl-4-(1-methylethylidene)-	586-62-9	1089	1088
**16**	12.48	3.49	Linalool	78-70-6	1100	1099
**17**	12.65	0.16	Nonanal	124-19-6	1105	1104
**18**	12.93	0.03	1,3,8-p-Menthatriene	18368-95-1	1112	1119
**19**	13.14	0.03	2-Cyclohexene-1-carboxaldehyde, 2,6,6-trimethyl-	432-24-6	1117	1116
**20**	13.35	0.05	2-Cyclohexen-1-ol, 1-methyl-4-(1-methylethyl)-, cis-	29803-82-5	1123	1122
**21**	13.94	0.03	2,6-Dimethyl-1,3,5,7-octatetraene, E,E-	460-01-5	1137	1131
**22**	14.30	0.05	Lilac aldehyde A	53447-46-4	1145	1145
**23**	14.37	0.18	Cyclohexanol, 1-methyl-4-(1-methylethenyl)-	138-97-4	1147	1153
**24**	14.66	0.07	Lilac aldehyde D	53447-47-5	1154	1169
**25**	14.77	0.07	Citronellal	106-23-0	1156	1153
**26**	15.22	0.01	p-Menth-8-en-1-ol, stereoisomer	138-87-4	1166	1161
**27**	15.79	0.70	Terpinen-4-ol	7299-40-3	1178	1177
**28**	16.17	0.02	Benzenemethanol, α,α,4-trimethyl-	1197-01-9	1186	1183
**29**	16.40	1.23	L-α-Terpineol	10482-56-1	1191	1189
**30**	16.78	0.04	1,3-Cyclohexadiene-1-carboxaldehyde, 2,6,6-trimethyl-	116-26-7	1198	1201
**31**	17.10	0.59	Decanal	112-31-2	1206	1206
**32**	17.65	0.45	2-Cyclohexen-1-ol, 2-methyl-5-(1-methylethenyl)-, cis-	2102-59-2	1220	1229
**33**	18.13	0.30	Citronellol	106-22-9	1231	1228
**34**	18.38	0.66	Benzene, 2-methoxy-4-methyl-1-(1-methylethyl)-	1076-56-8	1237	1235
**35**	18.73	0.03	D-Carvone	2244-16-8	1245	1246
**36**	19.29	0.08	Geraniol	106-24-1	1258	1257
**37**	19.56	0.03	2-Decenal, (E)-	3913-81-3	1264	1263
**38**	20.04	0.25	1-Cyclohexene-1-carboxaldehyde, 4-(1-methylethenyl)-	2111-75-3	1274	1272
**39**	20.80	0.05	p-Cymen-7-ol	536-60-7	1290	1289
**40**	20.99	1.21	Thymol	89-83-8	1294	1291
**41**	21.37	0.03	Phenol, 2-ethyl-4,5-dimethyl-	2219-78-5	1302	1305
**42**	21.79	0.42	2-Methoxy-4-vinylphenol	7786-61-0	1313	1317
**43**	21.92	0.08	2,4-Decadienal, (E,E)-	25152-84-5	1316	1317
**44**	22.82	0.09	Cyclohexene, 4-ethenyl-4-methyl-3-(1-methylethenyl)-1-(1-methylethyl)-, (3R-trans)-	20307-84-0	1338	1338
**45**	23.57	0.07	6-Octen-1-ol, 3,7-dimethyl-, acetate	150-84-5	1356	1354
**46**	24.05	0.06	2,6-Octadien-1-ol, 3,7-dimethyl-, acetate, (Z)-	141-12-8	1367	1364
**47**	24.23	0.06	n-Decanoic acid	334-48-5	1371	1373
**48**	24.42	0.03	Copaene	3856-25-5	1376	1376
**49**	24.86	0.04	Geranyl acetate	105-87-3	1385	1382
**50**	25.14	0.06	Cyclohexane, 1-ethenyl-1-methyl-2,4-bis(1-methylethenyl)-, [1S-(1α,2β,4β)]-	515-13-9	1392	1391
**51**	25.87	0.08	Dodecanal	112-54-9	1409	1409
**52**	26.20	0.01	Caryophyllene	87-44-5	1418	1419
**53**	26.83	0.01	γ-Elemene	29873-99-2	1434	1433
**54**	27.01	0.01	α-Guaiene	3691-12-1	1438	1439
**55**	27.60	0.05	Humulene	6753-98-6	1453	1454
**56**	28.72	0.14	Germacrene D	23986-74-5	1480	1481
**57**	28.96	0.03	trans-β-Ionone	79-77-6	1486	1486
**58**	29.16	0.02	δ-Selinene	28624-23-9	1490	1493
**59**	29.36	0.03	(1S,2E,6E,10R)-3,7,11,11-Tetramethylbicyclo [8.1.0] undeca-2,6-diene	24703-35-3	1495	1495
**60**	29.55	0.01	α-Muurolene	10208-80-7	1499	1499
**61**	29.68	0.02	Naphthalene, 1,2,3,5,6,7,8,8a-octahydro-1,8a-dimethyl-7-(1-methylethenyl)-, [1S-(1α,7α,8aα)]-	10219-75-7	1503	1499
**62**	29.92	0.06	α-Farnesene		1509	1508
**63**	30.45	0.06	Naphthalene, 1,2,4a,5,8,8a-hexahydro-4,7-dimethyl-1-(1-methylethyl)-, [1S-(1α.,4aβ,8aβ)]-	523-47-7	1523	1518
**64**	31.39	0.01	Cyclohexanemethanol, 4-ethenyl-α,α,4-trimethyl-3-(1-methylethenyl)-, [1R-(1α,3α,4β)]-	639-99-6	1548	1549
**65**	31.69	0.24	1,5-Cyclodecadiene, 1,5-dimethyl-8-(1-methylethylidene)-, (E,E)-	15423-57-1	1555	1557
**66**	32.13	0.19	Dodecanoic acid	143-07-7	1566	1568
**67**	32.50	0.02	1H-Cycloprop [e]azulen-7-ol, decahydro-1,1,7-trimethyl-4-methylene-, [1ar-(1aα,4aα,7β,7aβ,7bα)]-	6750-60-3	1576	1576
**68**	34.31	0.07	β-Asarone	5273-86-9	1623	1628
**69**	34.46	0.06	Isospathulenol	88395-46-4	1627	1638
**70**	34.55	0.01	2-Naphthalenemethanol, 1,2,3,4,4a,5,6,7-octahydro-α,α,4a,8-tetramethyl-, (2R-cis)-	1209-71-8	1630	1631
**71**	34.97	0.03	.tau.-Cadinol	5937-11-1	1641	1640
**72**	35.21	0.02	2-Naphthalenemethanol, decahydro-α,α,4a-trimethyl-8-methylene-, [2R-(2α,4aα,8aβ)]-	473-15-4	1648	1649
**73**	36.88	0.02	1-Naphthalenol, decahydro-1,4a-dimethyl-7-(1-methylethylidene)-, [1R-(1α	473-04-1	1693	1692
**74**	39.00	0.87	2,6,9,11-Dodecatetraenal, 2,6,10-trimethyl-, (E,E,E)-	17909-77-2	1754	1752
**75**	39.34	0.10	Tetradecanoic acid	544-63-8	1764	1768
**76**	44.54	0.01	5,9,13-Pentadecatrien-2-one, 6,10,14-trimethyl-, (E,E)-	1117-52-8	1919	1919
**77**	44.82	0.02	Hexadecanoic acid, methyl ester	112-39-0	1927	1926
**78**	45.96	0.41	n-Hexadecanoic acid	57-10-3	1962	1968
**79**	47.00	0.01	Hexadecanoic acid, ethyl ester	628-97-7	1994	1993
**80**	51.16	0.06	9,12-Octadecadienoic acid (Z,Z)-	60-33-3	2133	2133
**81**	67.94	0.64	2-Methylheptacosane	1561-00-8	2771	2762

### Active components of volatile oil

The chemical structures of all identified volatile oil components were retrieved from the PubChem database. Swiss ADME was used to screen for potential active components, identifying 39 candidates. Subsequently, Swiss Target Prediction was used to predict the targets of these components, resulting in 21 active compounds with 189 associated targets. Visualization was performed using Cytoscape, as shown below.

Among the active constituents of *Citrus reticulata* ‘Dahongpao’ essential oil, *D*-limonene is present in the highest concentration. However, due to its poor gastrointestinal absorption, it was not selected as a bioactive compound. Similarly, the screening of active ingredients based on criteria such as gastrointestinal absorption, ability to cross the blood-brain barrier, and drug-likeness does not necessarily correlate with compound abundance. Through Swiss Target Prediction, 21 active components were identified, including Cadinol, Spathulenol, α-Cyclocitral, cis-Linalool Oxide, γ-Eudesmol, and Elemol. Notably, compounds like linalool, thymol, cinnamic acid, myristic acid, and lauric acid have shown considerable pharmacological activity.

**Fig 2:**
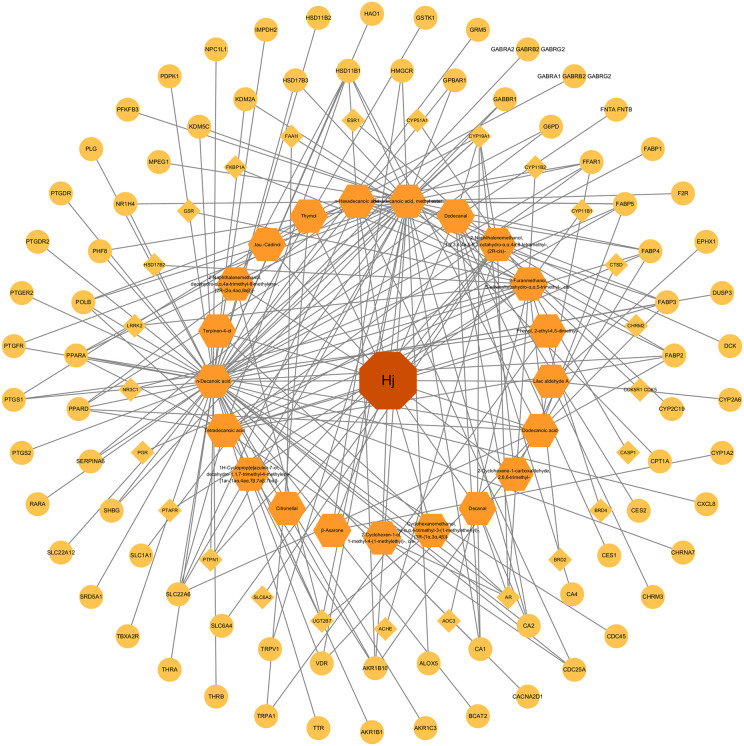
Compound–Target Network.

This network diagram (Fig 2) shows the interactions between compounds and their associated target proteins. The large central node represents *Citrus reticulata* ‘Dahongpao’ (Hj). The first inner layer of orange hexagonal nodes represents the 21 active components, and the yellow circular nodes represent the corresponding target proteins. The gray lines indicate the interactions between the compounds and proteins, forming a complex network.

### Diabetes-related targets and intersection targets

Diabetes-related targets were retrieved from the Gene Cards database, and filtering identified a total of 1,251 targets. Although *Citrus reticulata* ‘Dahongpao’ has 189 potential targets, only 102 remained after removing duplicates. The intersection between the disease and compound targets yielded 36 effective drug–disease overlapping targets.

**Fig 3:**
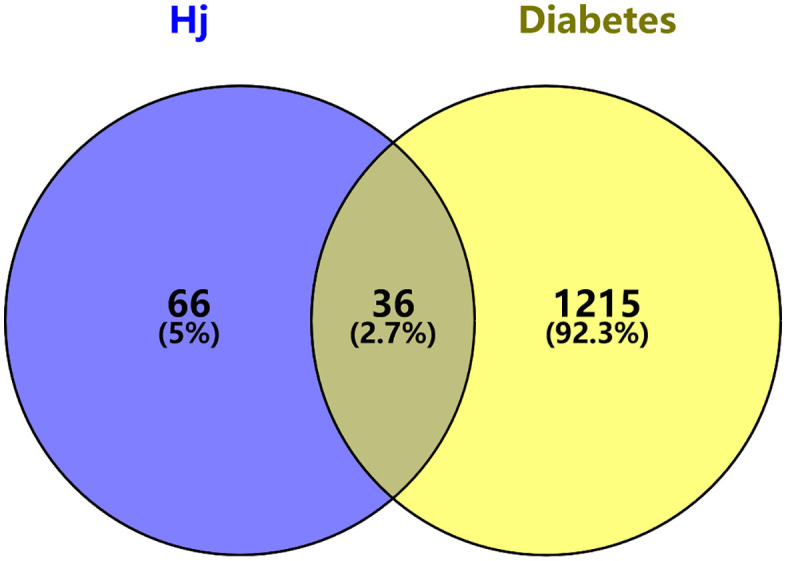
Drug–Disease Intersection Target Diagram.

**Fig 4:**
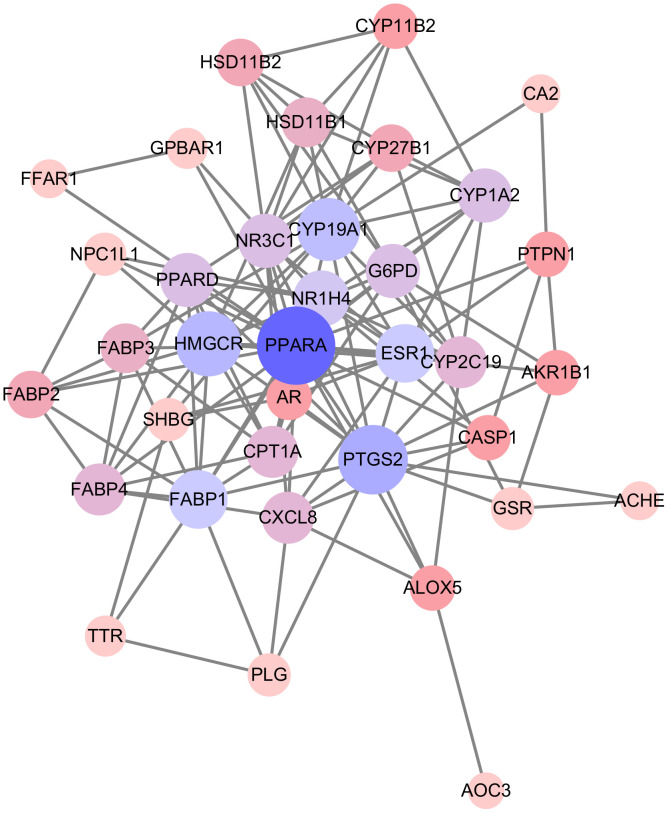
Core Target Protein Interaction Network.

The Venn diagram (Fig 3) shows the compound-related targets on one side and diabetes-related targets on the other, with the overlapping area representing the intersection. The Fig 4 displays the protein–protein interaction network of these 36 proteins, generated using the STRING database and optimized using Cytoscape. Different colored circles represent various proteins, and connecting lines represent their interactions. Darker colors indicate stronger associations.

At the center is PPARA (Peroxisome Proliferator-Activated Receptor Alpha), a nuclear receptor and ligand-dependent transcription factor. There are three PPAR isoforms: PPARα, PPARδ, and PPARγ. PPARα is highly expressed in the liver, heart, kidneys, and brown adipose tissue, regulating lipid metabolism, energy balance, and inflammatory responses. Its activation promotes fatty acid oxidation and ketogenesis, reduces triglyceride synthesis, and increases HDL(high density lipoprotein) cholesterol, thus improving lipid disorders and preventing cardiovascular diseases.

Moreover, PPARα exhibits anti-inflammatory effects and can mitigate the progression of atherosclerosis. Due to its broad biological functions, PPARα has become a crucial target in treating obesity, non-alcoholic fatty liver disease, type 2 diabetes, and other metabolic disorders.

Among the other targets, GLP1R is a G protein-coupled receptor expressed on intestinal cells that responds to GLP-1 stimulation, increasing insulin secretion and lowering blood glucose. INSR is a tyrosine kinase receptor widely present in the liver, adipose tissue, and muscle. It binds insulin and initiates downstream signaling pathways to increase glucose uptake and reduce blood sugar levels.

To further explore the potential of volatile oil in treating diabetes, the 36 overlapping targets were refined to 9 core targets based on their importance: FABP1, HMGCR, NR1H4, ESRRA, CYP19A1, NR3C1, G6PD, PPARA, and PTGS2.

Among the nine core targets identified—FABP1, HMGCR, NR1H4, ESRRA, CYP19A1, NR3C1, G6PD, PPARA, and PTGS2—several are closely associated with glucose and lipid metabolism. PPARA (Peroxisome Proliferator-Activated Receptor Alpha), for instance, plays a pivotal role in fatty acid oxidation and energy homeostasis and is recognized for its involvement in the pathogenesis of type 2 diabetes. Activation of PPARα has been reported to enhance insulin sensitivity and contribute to glycemic control [33]. NR1H4 (also known as FXR, Farnesoid X Receptor), aside from its regulatory role in bile acid metabolism, influences hepatic glucose production by modulating gluconeogenesis. Prior studies have shown that FXR can regulate insulin levels via Foxa2-mediated transcriptional control [34]. ESRRA is also implicated in maintaining glucose homeostasis. Disruption of ESRRA function has been shown to alter the expression of hepatic genes involved in lipid and carbohydrate metabolism in male rats, potentially modifying diabetes susceptibility [35]. CYP19A1 encodes the enzyme aromatase, which converts androgens into estrogens. Given estrogen’s favorable effect on insulin sensitivity, changes in CYP19A1 activity could indirectly impact diabetes risk. Although G6PD (Glucose-6-Phosphate Dehydrogenase) is primarily known for protecting red blood cells from oxidative stress, its role in glucose metabolism suggests that alterations in this pathway may influence overall metabolic health and diabetes development [36].

### GO and KEGG pathway analysis

**Fig 5:**
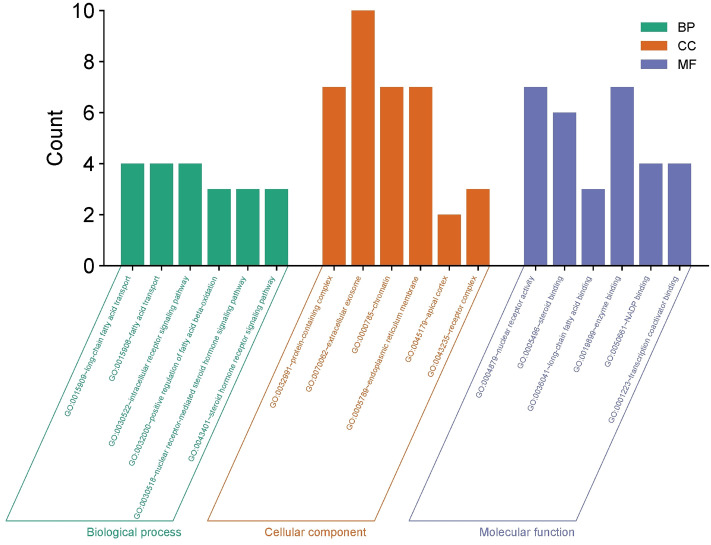
GO Analysis of Biological Process, Cellular Component, and Molecular Function.

Gene Ontology (GO) enrichment analysis was performed using the DAVID database, with results systematically filtered to highlight the six most relevant terms within each category: Biological Process (BP), Cellular Component (CC), and Molecular Function (MF). The findings, visualized via the “Microbioinformatics” platform (Fig 5), reveal functionally coherent mechanisms through which the volatile oil may alleviate diabetic symptoms.

In the BP category, significant enrichment was observed in processes including fatty acid transport, long-chain fatty acid transport, nuclear receptor-mediated signaling, and glucocorticoid receptor signaling. These terms are closely associated with lipid metabolic pathways known to be dysregulated in diabetes. Notably, ion transport—encompassing potassium and transmembrane ion movement—emerged as a key process, corroborating its essential role in insulin secretion from pancreatic β-cells and the maintenance of ion homeostasis. Disruptions in these processes are hallmarks of diabetic pathology, suggesting that the volatile oil may exert therapeutic effects by modulating ion channel activity and nuclear receptor signaling pathways.

At the CC level, enrichment was identified in exosomes, chromatin, endoplasmic reticulum membrane, and receptor complexes. These components are functionally significant in diabetes: exosomes participate in intercellular communication and hormone regulation; chromatin modifications suggest potential epigenetic influences on gene expression; the endoplasmic reticulum is central to protein synthesis and cellular stress response; and receptor complexes are critical for signal transduction, including insulin signaling. The prominence of cytoplasmic and mitochondrial components—key hubs for glucose and lipid metabolism—further supports the involvement of metabolic and energy-producing pathways affected by diabetes.

Within the MF category, enriched terms such as nuclear receptor activity, steroid binding, long-chain fatty acid binding, enzyme binding, NADP⁺ binding, and transcription activator binding indicate multifaceted mechanisms at the molecular level. Nuclear receptor activity and steroid binding imply direct modulation of receptors such as PPARs, which play established roles in glucose and lipid homeostasis. Concurrently, enzyme-binding and nucleotide-binding activities highlight the regulation of catalytic functions and energy metabolism, while lipid-binding proteins appear crucial for fatty acid transport. These molecular functions collectively underpin key diabetic mechanisms, including insulin signal transduction, energy metabolism, and transcriptional activation.

Overall, the GO analysis suggests that the volatile oil may ameliorate diabetic symptoms through a multi-level regulatory network involving nuclear receptor signaling, ion transport, epigenetic modulation, and metabolic enzyme activity. These insights not only help explain its pharmacological effects but also provide a theoretical foundation for developing volatile oil-based antidiabetic therapies.

**Fig 6:**
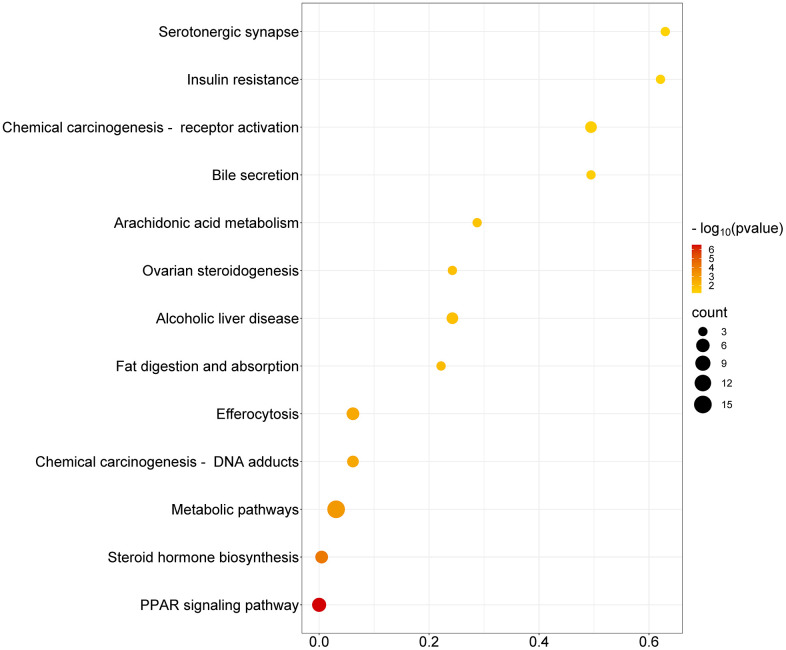
KEGG Pathway Enrichment Chart.

KEGG pathway enrichment analysis, conducted via the DAVID database and visualized using the Microbioinformatics platform, Fig 6 identified thirteen significantly enriched pathways (P-value < 0.05; see Supplementary S1 Table for FDR-adjusted values). Each pathway is represented in the scatter plot with its position, dot color, and size reflecting statistical significance and gene count metrics.Notably, the significant enrichment of the “Insulin resistance” pathway aligns with the potential of bioactive compounds in the extract to enhance insulin signaling (KEGG: hsa04910), potentially improving pancreatic β-cell functionality and promoting insulin secretion [37]. Similarly, the prominence of “Metabolic pathways” underscores a systemic regulatory role in glucose and lipid metabolism. Pathways such as “PPAR signaling” (KEGG: hsa03320) further support this mechanism, indicating that the facilitation of fatty acid oxidation and improved energy efficiency may alleviate insulin resistance [38].

However, “Insulin resistance” and “Serotonergic synapse” given their FDR values greater than 0.05 (see S1 Table), these findings should be considered exploratory and interpreted with caution. Therefore, we place greater emphasis on the more statistically robust pathways, particularly the PPAR signaling pathway, which demonstrates strong significance (FDR < 0.01) and is mechanistically well-supported by our network pharmacology and docking results.

Other relevant pathways, including “Chemical carcinogenesis – receptor activation”, “Bile secretion”, and “Arachidonic acid metabolism”, also show substantial gene counts and significance. These suggest additional therapeutic effects: suppression of carcinogenic processes, improved bile flow and lipid digestion, and modulation of inflammatory responses—factors often dysregulated in diabetic conditions. In particular, arachidonic acid metabolism (KEGG: hsa00590) may contribute to resolving chronic inflammation associated with insulin resistance, while modulation of fat digestion and absorption (KEGG: hsa04975) offers broader metabolic benefits [39].

### Molecular docking

Protein structures corresponding to the core targets were obtained from the PDB database. Based on gene counts of active components, literature reports on *Citrus* bioactive compounds, and relevance to diabetes, five small molecules were selected for molecular docking:Linalool, Dodecanoic acid, n-Hexadecanoic acid, Tetradecanoic acid, and Thymol.

A total of 45 docking combinations were conducted, and the 10 with the best binding energies are shown in the Table 2.

**Table 2:** Binding energy data of 10 groups of molecular docking.

Protein	PDB ID	Molecule	Binding energy (kcal/mol)
**FABP1**	2F73	Thymol	−6.8
**PTGS2**	5F19	n-Hexadecanoic acid	−6.7
**FXR**	1OSH	Thymol	−6.6
**PTGS2**	5F19	Thymol	−6.5
**FABP1**	2F73	Dodecanoic acid	−6.4
**PTGS2**	5F19	Dodecanoic acid	−6.2
**PTGS2**	5F19	Linalool	−6.2
**ESRRA**	3D24	Thymol	−6
**CYP19A1**	3S79	Thymol	−5.9
**G6PD**	60E8	Dodecanoic acid	−5.9

**Fig 7:**
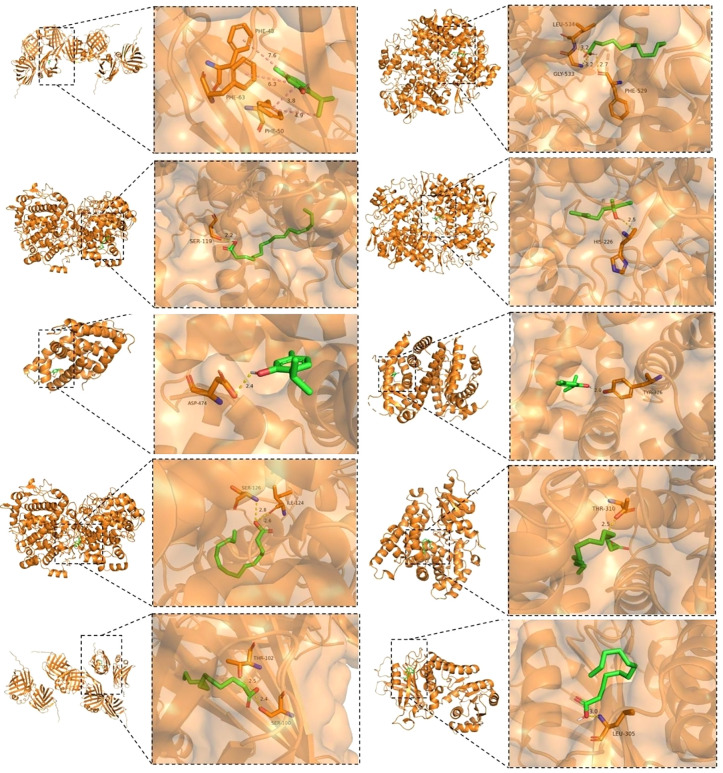
Molecular Docking Visualization.

Representative docking results are visualized in Fig 7, which illustrates both the overall binding conformation and detailed hydrogen-bond interactions between ligands and proteins. For instance, Thymol formed a hydrogen bond with THR-102 in FABP1 (bond length: 2.5 Å), indicating stable binding within the protein’s active site.

The docking analysis revealed high-affinity interactions between these compounds and diabetes-related targets, corroborating and extending previous findings. Thymol exhibited strong binding to FABP1 (−6.8 kcal·mol ⁻ ¹; PDB:2F73) [40], consistent with its reported role in lipid transport modulation [41,42]. Its notable affinity for FXR (−6.6 kcal·mol ⁻ ¹; PDB:1OSH) further suggests a potential role in bile acid and glucose metabolic crosstalk [43]. Similarly, n-Hexadecanoic acid showed favorable binding to PTGS2 (−6.7 kcal·mol ⁻ ¹), supporting possible anti-inflammatory effects relevant to insulin resistance.

While these results indicate robust binding under static conditions, it is important to note that molecular docking alone cannot fully capture dynamic interaction behaviors—such as the stability of n-hexadecanoic acid within the PTGS2 binding pocket under physiological conditions. Thus, these computational findings warrant further validation through functional assays or dynamic simulation studies.

Overall, the molecular docking results support a multi-target mechanism through which the volatile oil’s components may regulate diabetic pathways, including lipid metabolism, inflammatory response, and nuclear receptor signaling

### Molecular dynamics (MD)

#### Stability analysis.

**Fig 8:**
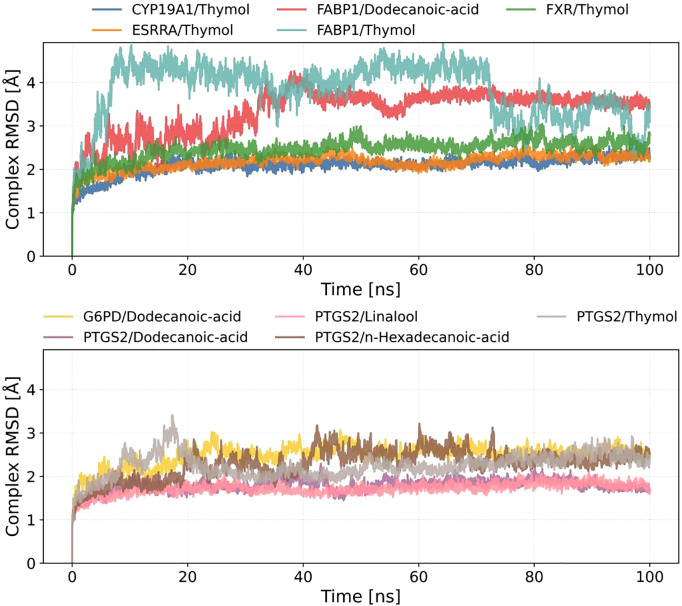
Time-dependent root mean square deviation (RMSD) of protein-ligand complexes during molecular dynamics simulations.

To evaluate the conformational stability of ligand-protein complexes, 100-ns molecular dynamics (MD) simulations were performed on ten systems, and the RMSD trajectories were analyzed (Fig 8). The results indicated that most systems reached equilibrium within the initial 20 ns (RMSD < 2.5 Å), followed by reduced fluctuations, suggesting structural convergence.

Notably, FABP1/Dodecanoic-acid and FABP1/Thymol exhibited pronounced RMSD fluctuations, with average values of 3.5 Å and 4.0 Å, respectively. Transient spikes (>1.0 Å) persisted throughout the simulations, implying potential conformational rearrangements or enhanced local flexibility of the ligands within the binding pocket. In contrast, systems such as CYP19A1/Thymol, ESRRA/Thymol, and FXR/Thymol demonstrated high structural rigidity, maintaining RMSD values below 2.5 Å with low standard deviations (SD < 0.3 Å), indicative of stable binding modes.

For PTGS2-related complexes (e.g., PTGS2/Linalool, PTGS2/Dodecanoic-acid, PTGS2/n-Hexadecanoic-acid, PTGS2/Thymol), moderate RMSD fluctuations (2.0–3.0 Å) were observed. Fatty acid ligands (e.g., Dodecanoic-acid) showed slightly higher RMSD values compared to terpenoids (Linalool) or phenolics (Thymol), likely attributable to the conformational flexibility of their aliphatic chains.

These findings highlight the critical role of ligand chemical architecture in modulating complex stability. Subsequent analyses of binding free energy and hydrogen bond dynamics will further elucidate the mechanistic links between conformational fluctuations and intermolecular interactions.

**Fig 9:**
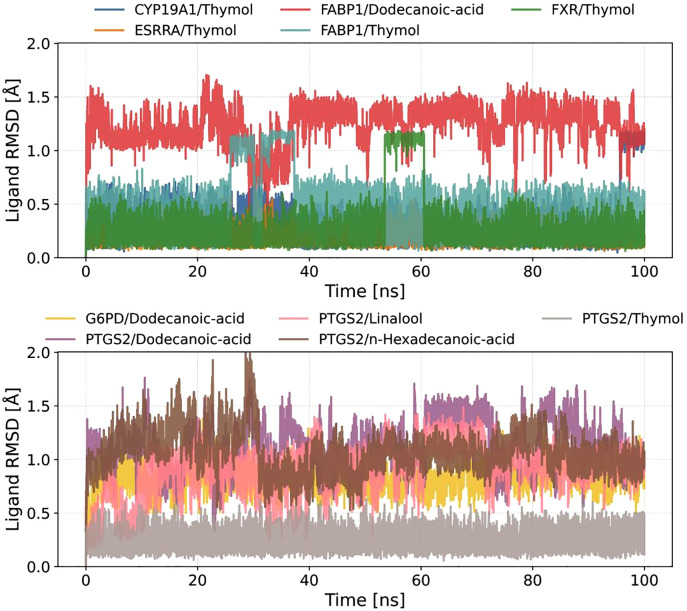
Time-dependent root mean square deviation (RMSD) of ligands during molecular dynamics simulations.

To investigate the conformational stability of ligands within binding pockets, the RMSD trajectories of ligands were analyzed over 100-ns MD simulations (Fig 9). The majority of ligands exhibited low RMSD values (<1.5 Å), indicating high conformational rigidity in their bound states.

Notably, FABP1/Dodecanoic-acid displayed the most pronounced RMSD fluctuations (1.0–1.8 Å), with abrupt spikes (>0.8 Å ΔRMSD at 30 ns and 75 ns), suggesting dynamic repositioning of its aliphatic chain to accommodate the binding pocket. For PTGS2/n-Hexadecanoic-acid, the RMSD progressively increased during the initial 50 ns (0.8 → 1.5 Å) before stabilizing at ~1.2 Å, indicative of a conformational relaxation phase.

In contrast, phenolic ligands (e.g., CYP19A1/Thymol, ESRRA/Thymol, FXR/Thymol) maintained exceptionally low RMSD values (<0.5 Å, SD < 0.1 Å), reflecting the rigid anchoring of their aromatic rings within the binding sites. The terpenoid PTGS2/Linalool and short-chain fatty acid PTGS2/Dodecanoic-acid exhibited moderate fluctuations (0.5–1.0 Å), likely attributable to balanced flexibility and transient hydrogen bond interactions.

These results underscore the critical role of ligand chemistry in binding stability: fatty acids with extended chains undergo conformational adaptations, while aromatic and terpenoid ligands achieve stability through structural rigidity. This structural insight lays a foundation for further exploration of energy landscapes and interaction mechanisms.

**Fig 10:**
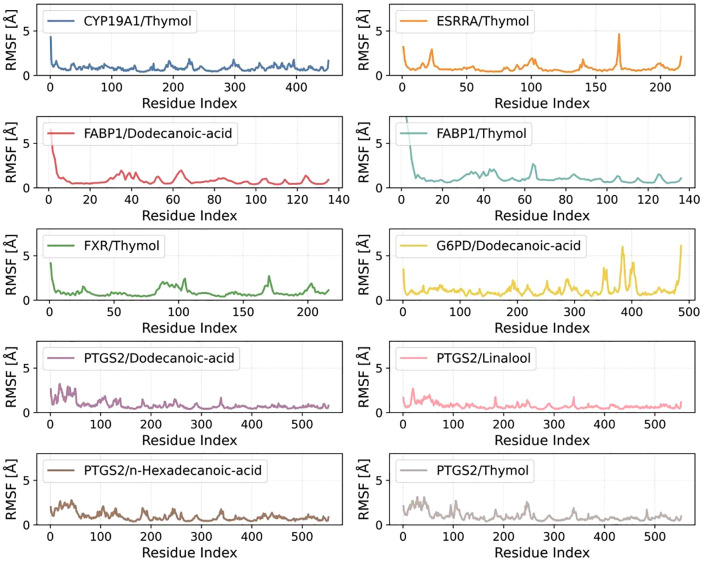
Root mean square fluctuation (RMSF) calculated based on molecular dynamics simulation trajectory.

Fig 10 illustrates the RMSF profiles of backbone residues across ten protein–ligand complexes throughout 100-ns, aiming to assess the differential local flexibility upon ligand binding.

Among all complexes, CYP19A1/Thymol, ESRRA/Thymol, and FXR/Thymol consistently exhibited low RMSF values, with most residues fluctuating below 2.0 Å. This pattern suggests high conformational rigidity and a stable global fold in response to Thymol binding. In particular, CYP19A1/Thymol showed only minor fluctuations at the N-terminal region, indicating that Thymol contributes to structural stabilization of the protein framework.

In contrast, FABP1-bound systems, especially FABP1/Dodecanoic-acid and FABP1/Thymol, displayed significantly elevated RMSF values in the N-terminal and central regions, with peaks reaching 4–5 Å. Such deviations are indicative of conformational rearrangements or increased local flexibility triggered by ligand accommodation. Notably, Dodecanoic acid induced more extensive fluctuations than Thymol, likely due to the inherent flexibility of its aliphatic chain.

The G6PD/Dodecanoic-acid complex demonstrated a marked increase in RMSF at the C-terminal region, exceeding 6 Å, which may reflect ligand-induced loosening or global shifts in protein conformation. A similar trend was observed in PTGS2-associated systems (e.g., PTGS2/Dodecanoic-acid, PTGS2/n-Hexadecanoic-acid, PTGS2/Thymol, and PTGS2/Linalool), where specific segments exhibited moderate-to-high fluctuations. Fatty acid ligands in particular induced greater flexibility, possibly arising from increased internal torsions or dynamic hydrophobic tail movements.

Collectively, aromatic ligands such as Thymol consistently correlated with reduced protein flexibility, underscoring their potential role in stabilizing protein conformations. Conversely, fatty acid-based ligands promoted greater local dynamics, highlighting a structure–flexibility relationship that could have implications for ligand binding mechanisms and functional modulation. These observations provide a mechanistic basis for further energetic and structural characterization of these systems.

**Fig 11:**
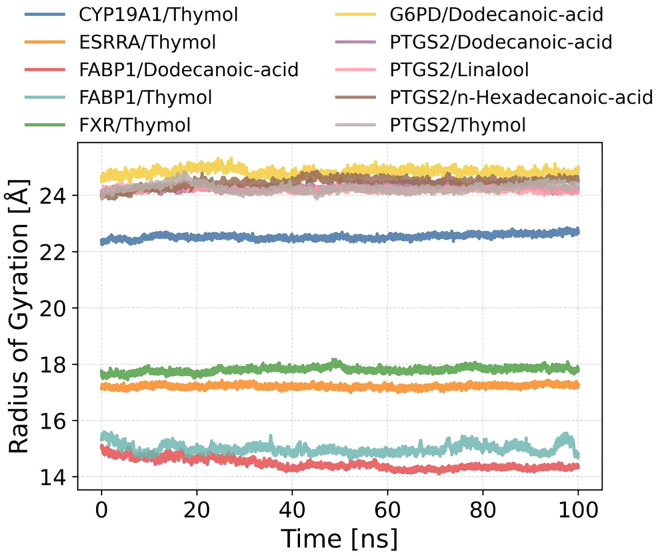
Time-dependent radius of gyration (Rg) profiles of protein–ligand complexes during 100-ns molecular dynamics simulations.

Fig 11 presents the time-dependent radius of gyration (Rg) profiles of the protein–ligand complexes obtained from 100-ns molecular dynamics simulations, aiming to evaluate their global compactness and conformational stability.

Overall, all complexes exhibited minimal fluctuations in Rg throughout the simulation, without any abrupt increases or long-term drift. This indicates that the systems remained structurally stable and did not undergo significant conformational loosening or global rearrangements. Notably, complexes such as CYP19A1/Thymol, FXR/Thymol, and ESRRA/Thymol maintained consistent Rg values around 22.5 Å, 17.8 Å, and 17.3 Å, respectively. The near-flat trajectories reflect well-preserved tertiary structures and high structural integrity upon ligand binding.

In comparison, the FABP1-based systems (FABP1/Thymol and FABP1/Dodecanoic-acid) displayed lower Rg values of approximately 15.5 Å and 15.0 Å, respectively, which is consistent with the relatively smaller size of the FABP1 protein. The Rg traces of both systems were notably stable, with no substantial deviations, suggesting minimal structural disruption induced by ligand interaction.

Furthermore, the lower panel illustrates that PTGS2-associated complexes—whether bound to Thymol, Linalool, Dodecanoic-acid, or n-Hexadecanoic-acid—as well as G6PD/Dodecanoic-acid, consistently maintained Rg values around 24.5 Å. This uniformity across different systems highlights their comparable compactness and reinforces the notion of structural stability under simulation conditions.Collectively, the MD simulations resolved key limitations of static docking by providing temporal insights into complex stability and ligand adaptability. Crucially, Thymol complexes maintained structural rigidity (RMSD < 2.5 Å), confirming stable binding, while fatty acid ligands induced adaptive conformational changes (RMSD 3.5–4.0 Å; RMSF peaks 4–5 Å) that optimized hydrophobic packing—behavior undetectable by docking alone. Hydrogen bond analysis further differentiated the binding mechanisms: persistent H-bonds (3–5) stabilized fatty acid-PTGS2 complexes, whereas Thymol relied predominantly on hydrophobic interactions (≤2 H-bonds), consistent with its transient interaction with FABP1/THR102 (2.5 Å). These dynamic profiles not only validate the docking predictions but also elucidate ligand-specific binding modes that are critical for target modulation [44].

#### MM-GBSA binding free energy results.

**Fig 12:**
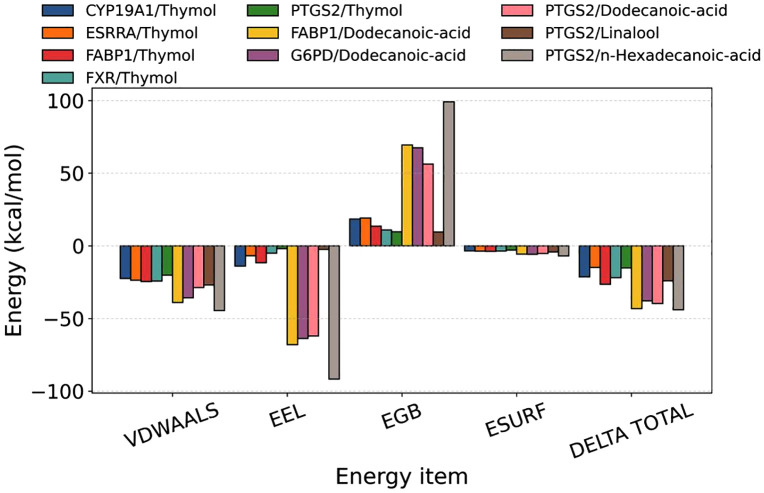
Decomposition of binding free energy and contributions of individual energy terms for protein-ligand complexes.

To quantitatively assess the binding affinities between ligands and their target proteins, MM-GBSA calculations were performed based on the molecular dynamics trajectories (Table 3, Fig 12). The binding free energy (ΔGbind) values for all complexes were negative, suggesting that each ligand can spontaneously associate with its corresponding protein.

**Table 3:** Binding free energies and energy components predicted by MM/GBSA (kcal/mol).

System	ΔE_vdW_	ΔE_elec_	ΔG_GB_	ΔG_SA_	ΔG_bind_
**CYP19A1/Thymol**	−22.40 ± 1.00	−13.99 ± 1.32	18.49 ± 1.30	−3.43 ± 0.12	−21.33 ± 1.37
**ESRRA/Thymol**	−23.69 ± 1.19	−6.80 ± 1.78	19.20 ± 1.39	−3.59 ± 0.10	−14.87 ± 2.22
**FABP1/Thymol**	−24.59 ± 1.80	−11.65 ± 1.81	13.58 ± 0.79	−3.80 ± 0.13	−26.45 ± 1.34
**FXR/Thymol**	−24.26 ± 2.01	−5.03 ± 1.79	10.91 ± 0.75	−3.51 ± 0.12	−21.89 ± 0.92
**PTGS2/Thymol**	−20.13 ± 1.00	−1.88 ± 1.82	9.76 ± 1.40	−2.88 ± 0.13	−15.13 ± 1.22
**FABP1/Dodecanoic-acid**	−38.99 ± 1.23	−68.04 ± 5.38	69.51 ± 5.60	−5.75 ± 0.14	−43.26 ± 2.53
**G6PD/Dodecanoic-acid**	−35.80 ± 1.82	−63.79 ± 6.13	67.50 ± 4.20	−5.77 ± 0.08	−37.86 ± 4.18
**PTGS2/Dodecanoic-acid**	−28.72 ± 4.16	−62.03 ± 5.87	56.31 ± 4.07	−5.23 ± 0.11	−39.67 ± 2.81
**PTGS2/Linalool**	−26.90 ± 1.42	−2.46 ± 2.22	9.54 ± 1.07	−4.22 ± 0.08	−24.04 ± 2.06
**PTGS2/n-Hexadecanoic-acid**	−44.49 ± 2.98	−91.71 ± 7.85	99.20 ± 7.45	−6.92 ± 0.14	−43.93 ± 2.16

ΔE_vdW_: van der Waals energy. ΔE_elec_: electrostatic energy. ΔG_GB_: electrostatic contribution to solvation. ΔG_SA_: non-polar contribution to solvation. ΔG_bind_: binding free energy.

The MM-GBSA results revealed a clear hierarchy of binding affinities. Notably, the complexes involving fatty acid ligands, FABP1/Dodecanoic-acid and PTGS2/n-Hexadecanoic-acid, exhibited the most favorable ΔGbind values (below −40.0 kcal/mol), indicating exceptionally strong and spontaneous binding. In contrast, the Thymol complexes, particularly with ESRRA and PTGS2, showed relatively weaker, though still significant, binding affinities. Decomposition of the energy contributions provided mechanistic insights: the strong binding of fatty acids was driven by highly favorable van der Waals and electrostatic interactions, which overcame a large desolvation penalty. This suggests extensive hydrophobic contacts and potential polar interactions within the binding pockets. For Thymol, van der Waals forces were the dominant driving force. These energetic profiles corroborate the potential of these compounds to effectively modulate their respective targets—fatty acids in direct lipid handling and inflammation, and Thymol in nuclear receptor-mediated metabolic regulation.

Decomposition of energy contributions revealed that van der Waals (ΔE_vdW_) and electrostatic (ΔE_elec_) interactions were the primary driving forces for binding, whereas solvation energies, particularly the polar component (ΔG_GB_), generally acted unfavorably and countered complex formation to varying extents.

**Fig 13:**
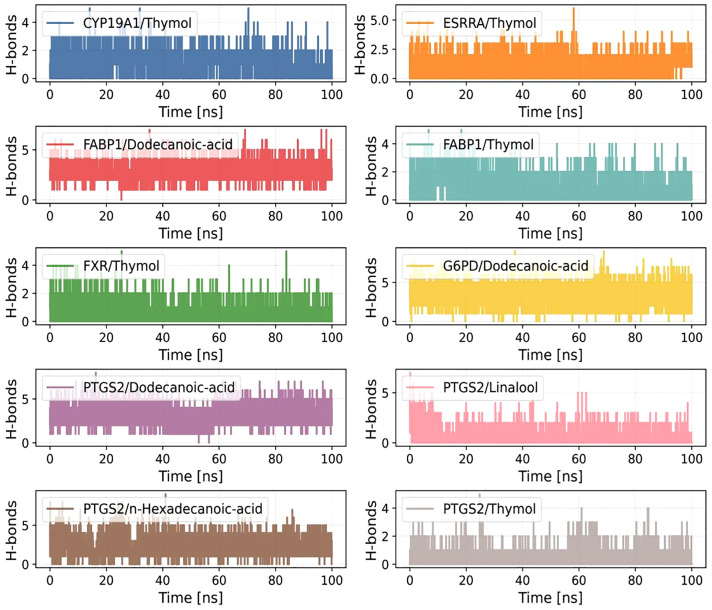
Changes in the number of hydrogen bonds between small molecules and proteins during molecular dynamics simulation.

Fig 13 illustrates the time-dependent hydrogen bond profiles observed throughout the 100-ns MD simulations. Marked differences in hydrogen bond counts were evident across complexes. The FABP1/Dodecanoic-acid, PTGS2/Dodecanoic-acid, and PTGS2/n-Hexadecanoic-acid systems maintained consistently higher hydrogen bond numbers, typically ranging from 3 to 5 with relatively minor fluctuations, implying tight and stable interactions at the protein–ligand interface.

In contrast, Thymol-based complexes such as CYP19A1/Thymol, FXR/Thymol, and PTGS2/Thymol formed fewer hydrogen bonds, often fewer than two throughout the simulation, and occasionally exhibited complete absence of hydrogen bonding, suggesting that hydrophobic or van der Waals forces might dominate in maintaining their binding stability. Intermediate hydrogen bond dynamics were observed in PTGS2/Linalool and FABP1/Thymol, with bond numbers fluctuating between 1 and 3, reflecting a balance between conformational flexibility and transient non-covalent interactions.

Critically, integration of the energy data with the earlier structural stability metrics (RMSF, RMSD) provides a mechanistic link between conformational stability and biological function. The low RMSF values (<2.0 Å) observed in Thymol-bound nuclear receptors (e.g., FXR, PPARA) suggest ligand-induced stabilization of their DNA-binding domains, which may enhance transcriptional regulation relevant to glucose and lipid metabolism [33,34]. Conversely, the persistent hydrogen bonding and high binding affinity of fatty acid ligands (e.g., n-Hexadecanoic acid) within PTGS2 indicate potential prolonged suppression of prostaglandin synthesis—a key mechanism in mitigating diabetes-related inflammation [43].

In summary, the MM-GBSA results establish a robust energetic hierarchy among the complexes and clarify the intermolecular forces governing ligand binding. When combined with structural dynamics data, they provide a multi-scale mechanistic understanding of how specific compounds in the volatile oil may modulate target proteins to alleviate diabetic pathology.

## Conclusions

This integrative study elucidates the multi-target anti-diabetic mechanisms of *Citrus reticulata* ‘Dahongpao’ through a synergistic computational-experimental framework. GC-MS analysis identified 82 constituents (96.61% coverage), with *D*-limonene (62.48%), γ-terpinene (9.82%), and thymol (1.21%) as major components. Network pharmacology revealed 36 diabetes-related targets, with nine core targets (PPARA, FXR, PTGS2, FABP1, HMGCR, ESRRA, CYP19A1, NR3C1, G6PD) implicated in nuclear receptor signaling, lipid metabolism, and inflammatory pathways. Molecular docking prioritized high-affinity interactions, notably thymol binding to FABP1 (−6.8 kcal/mol) and n-hexadecanoic acid to PTGS2 (−6.7 kcal/mol).

Critical validation via 100-ns molecular dynamics simulations demonstrated structural stability for key complexes (e.g., CYP19A1/thymol, RMSD < 2.5 Å) and revealed ligand-specific dynamic behaviors: Fatty acids (dodecanoic/n-hexadecanoic acid) induced adaptive flexibility in FABP1/PTGS2 (RMSF 4–6 Å), optimizing hydrophobic packing. Thymol stabilized nuclear receptors (PPARA/FXR, RMSF < 2.0 Å) through rigid binding.

MM-GBSA quantified robust binding energetics for FABP1/dodecanoic acid (−43.26 kcal/mol) and PTGS2/n-hexadecanoic acid (−43.93 kcal/mol), driven predominantly by van der Waals forces (ΔE_vdW_). Hydrogen bond analysis further differentiated mechanisms: persistent polar interactions stabilized fatty acid complexes (3–5 bonds), while thymol relied on hydrophobic dominance (≤2 bonds).

These findings establish that essential oil combats diabetes through synergistic multi-target modulation: Nuclear receptor stabilization (PPARA/FXR) enhances insulin sensitivity and lipid homeostasis. PTGS2 inhibition disrupts inflammatory cascades driving insulin resistance. FABP1-mediated fatty acid transport resolves lipid dysregulation.

The work pioneers a dynamic validation paradigm for natural product research, integrating phytochemistry, multi-target networking, and atomistic simulations to bridge computational predictions with mechanistic plausibility. Future studies should prioritize in vivo validation of dose-dependent synergies and address batch variability in essential oil composition. This framework accelerates the development of evidence-based plant-derived therapeutics for metabolic disorders.

## Supporting information

S1 Table Supplementary data of KEGG enrichment analysis for the potential targets of Chongqing Citrus reticulata ‘Dahongpao’ volatile oil. (XLSX)
